# Exploring the therapeutic potential of isoorientin in the treatment of osteoporosis: a study using network pharmacology and experimental validation

**DOI:** 10.1186/s10020-024-00799-7

**Published:** 2024-02-20

**Authors:** Bo Zhang, Zechao Qu, Hua Hui, Baorong He, Dong Wang, Yong Zhang, Yiwei Zhao, Jingjun Zhang, Liang Yan

**Affiliations:** 1 Department of Spine Surgery, Honghui Hospital, Xi’an Jiaotong University, Xi’an, 710054 China; 2 Health Science Center, Xi’an Jiaotong University, Xi’an, China

**Keywords:** Osteoclast, Receptor activator of nuclear factor-κB ligand (RANKL), Reactive oxygen species (ROS), Isoorientin (ISO), Nuclear factor erythroid 2‐related factor 2(Nrf2)

## Abstract

**Background:**

Isoorientin (ISO) is a glycosylated flavonoid with antitumor, anti-inflammatory, and antioxidant properties. However, its effects on bone metabolism remain largely unknown.

**Methods:**

In this study, we aimed to investigate the effects of ISO on receptor activator of nuclear factor-κB ligand (RANKL)-induced osteoclast formation in vitro and bone loss in post-ovariectomy (OVX) rats, as well as to elucidate the underlying mechanism. First, network pharmacology analysis indicated that MAPK1 and AKT1 may be potential therapeutic targets of ISO and that ISO has potential regulatory effects on the mitogen-activated protein kinase (MAPK) and phosphoinositide 3-kinase (PI3K)/protein kinase B (AKT) pathways, as well as oxidative stress. ISO was added to RAW264.7 cells stimulated by RANKL, and its effects on osteoclast differentiation were evaluated using tartrate‐resistant acid phosphatase (TRAP) staining, TRAP activity measurement, and F-actin ring analysis. Reactive oxygen species (ROS) production in osteoclasts was detected using a ROS assay kit. The effects of ISO on RANKL-triggered molecular cascade response were further investigated by Western blotting, quantitative real-time polymerase chain reaction, and immunofluorescence staining. In addition, the therapeutic effects of ISO were evaluated in vivo.

**Results:**

ISO inhibited osteoclastogenesis in a time- and concentration-dependent manner. Mechanistically, ISO downregulated the expression of the main transcription factor for osteoclast differentiation by inhibiting MAPK and PI3K/AKT1 signaling pathways. Moreover, ISO exhibited protective effects in OVX-induced bone loss rats. This was consistent with the results derived from network pharmacology.

**Conclusion:**

Our findings suggest a potential therapeutic utility of ISO in the management of osteoclast-associated bone diseases, including osteoporosis.

## Introduction

Bone formation and resorption are tightly regulated processes that are primarily balanced by the activities of osteoclasts and osteoblasts (Elson et al. 2022). Osteoclasts (OCs) are multinucleated giant cells responsible for bone resorption and essential for normal bone health (Park-Min et al. 2022). Excessive osteoclast activity can lead to progressive bone loss, resulting in osteoporosis (OP), bone fragility, and fractures, which pose serious threats to human health. Currently, bisphosphonates are the most commonly used anti-OP drugs; however, they are associated with adverse events that limit their long-term use (Gao et al. 2022). Therefore, identifying safe and effective natural plant derivatives that inhibit the proliferation and differentiation of OCs as anti-OP treatment is important. Numerous studies have demonstrated the therapeutic potential of natural plant derivatives for OP (Nannan et al. 2023; Nor Muhamad et al. 2022; Qu et al. 2022a, b).

Network pharmacology merges disciplines like systems biology and bioinformatics and used to identify targets, functions, and its key feature is the integration of computational and experimental methods to explore action targets of traditional Chinese medicine and its compounds (Hopkins 2008; Wang et al. 2021). This approach aids in discovering new drug targets and understanding drug action mechanisms through biomolecular networks of diseases and syndromes. Network pharmacology has shifted the research focus from the traditional “drug-single target” model to a more comprehensive “drug-disease multi target gene-signaling pathway” paradigm, enhancing drug efficacy prediction and aiding in optimizing clinical drugs and understanding traditional drug mechanisms (Huang et al. 2014).

Isoorientin (3′,4′,5,7-tetrahydroxy-6-C-glucopyranosyl flavone, ISO) is a naturally occurring C-glycosyl flavonoid that is widely found in Chinese herbs and foods such as He Shou Wu, rooibos tea, corn, and buckwheat (Ziqubu et al. 2020). Previous studies have demonstrated its strong antioxidant and free radical scavenging effects and its anti-inflammatory and anti-tumor properties (Anilkumar et al. 2017; Xu et al. 2020; Yao et al. 2012; Yuan et al. 2014). Despite numerous reports on the biological activity of ISO, its effects on OCs remain unclear. The RAW264.7 cell line, derived from an Abelson murine leukemia virus-induced tumor, is easy to culture and can induce large numbers of OCs. Recently, it has been increasingly employed as a cellular model to study OCs because of its promising properties in macrophage-mediated immunity, metabolism, and phagocytosis (Agidigbi et al. 2019; Kitaura et al. 2020; Lorenzo 2017). Therefore, in this study, we combined network pharmacology and in vitro/in vivo experiments using RAW264.7 cells to assess the effect of ISO on osteoclast formation and to elucidate the associated molecular mechanisms.

## Materials and methods

### Reagents

ISO was purchased from MedChem Express (Monmouth Junction, NJ, USA), dissolved in dimethyl sulfoxide (DMSO; Beyotime) at the stock concentration of 20 mM, and diluted with culture medium to form working concentrations prior to use. Recombinant human RANKL was obtained from PeproTech EC, Ltd. Minimum Essential Medium Alpha (α-MEM), Dulbecco’s modified Eagle’s medium (DMEM), and fetal bovine serum (FBS) were purchased from Gibco. The CCK8 assay kit and tartrate‐resistant acid phosphatase (TRAP) assay kit were obtained from Beyotime. The TRAP staining kit was provided by Sigma‐Aldrich. The primary antibodies of NFATc1 and c-Fos were acquired from ABclonal. The primary antibodies for β-Actin, P38, phospho-P38, extracellular signal-egulated kinase (ERK), phospho-ERK, c-Jun N-terminal kinase (JNK), phospho-JNK, Nuclear factor erythroid 2‐related factor 2 (Nrf2), heme oxygenase‐1 (HO-1), catalase (CAT), and γ‐glutamyl cysteine synthetase catalytic subunit (GCLC) were acquired from Beyotime. The primary antibodies for phosphoinositide 3-kinase (PI3K), phospho-PI3K, Protein Kinase B α (AKT1), and phospho-AKT1 were purchased from Cell Signaling Technology.

### Potential targets intersection of ISO with OP

To reveal the multiple potential targets of ISO on OP and its mechanism of inhibition of osteoclast differentiation, network pharmacology was applied to this study. First, the standard structures of ISO were obtained from PubChem (https://pubchem.ncbi.nlm.nih.gov). Then, the Swiss Target Prediction (http://www.swisstargetprediction.ch/), STITCH database (http://stitch.embl.de) and PharmMapper (http://www.lilab-ecust.cn/pharmmapper/) for searching and screening relevant targets of ISO. The keyword “osteoporosis” was used in the Gene Cards (https://genecards.weizmann.ac.il/v3/), the OMIM Database (https://www.omim.org/) and the Therapeutic Target Database (TTD, http://db.idrblab.net/ttd/) were used to collect disease targets. UniProtKB (https://www.uniprot.org/) was applied to obtain the names of standard targets with “Homo sapiens” as the selected organism. The Venn online platform (http://bioinformatics.psb.ugent.be/webtools/Venn/) was used to intersect the active ingredient targets and disease targets retrieved from the above databases. This yielded the common target of the active ingredient and the disease, i.e., the target for the prevention and treatment of OP with ISO.

### Protein–protein interaction (PPI) network construction

The 134 common targets were uploaded to the STRING database to obtain the network relation of target interaction, with a minimum required interaction score of 0.4 (Gong et al. 2021). All data were imported into Cytoscape. Cytoscape 3.8.2 software was used to construct PPI networks.

### Enrichment analysis of gene ontology (GO) and Kyoto encyclopedia of genes and genomes (KEGG) pathways and signaling pathway-target networks

To convert the drug–disease common target into Entrez ID, we installed the Bioconductor package “org.Hs.eg.db” in R software and ran it. After that, the “ClusterProfiler” package was installed in R software, and enrichment analysis of GO and KEGG was carried out according to the transformed entrez ID, with p < 0.05. The results were output in the form of bar chart and bubble chart, respectively. We also created the signaling pathway-targeting network with Cytoscape software.

### Molecular docking between core targets and ISO

The structure files of ISO and proteins were downloaded from the PubChem database (https://pubchem.ncbi.nlm.nih.gov/) and the RCSB PDB protein database (https://www.rcsb.org/), respectively, and converted to mol2 format using OpenBabel software (version 3.1.1). After water removal, proligand removal, hydrogenation, and charge calculation, the data were saved as a PDBQT file. The target proteins were receptors, and the small molecules were ligands. AutoDock Vina 1.1.2 was used to dock molecules with proteins, and visualized using PYMOL software (version 2.3.0.).

### Cell culture and cell viability assays

RAW 264.7 mouse monocyte/macrophage cell lineage which was obtained from American Type Culture Collection (ATCC, Manassas, VA) was incubated in DMEM medium supplemented with 10% FBS. The cells were incubated in a cell incubator at a culture temperature of 37° C and a CO_2_ concentration of 5%. In this study, the CCK-8 assay was used to determine cell viability. RAW264.7 cells were seeded in 96-well plates at a density of 4 × 10^3^ cells/well. Cells were then exposed to varying concentrations of ISO (20, 40, 60, and 100 μM) to evaluate cytotoxic effects over 48 and 96-h periods. After adding 10 μL of CCK-8 solution to each well, the cell plates were subsequently incubated in the incubator for 2 h. The absorbance (optical density, OD) was measured at 450 nm using a microplate reader (EL × 800, USA).

### TRAP staining and TRAP activity measurement

RAW264.7 cells were seeded in 96-well plates and cultured in DMEM supplemented with 10% FBS and allowed to adhere overnight. The next day, the cells adhered to the bottom of the wells were cultured in complete α-MEM containing 50 ng/mL RANKL and treated with or without different concentrations of ISO (20, 40, 60, and 100 μM) for 5 days. The medium was replaced every other day. Cells were stained according to the TRAP instructions. OCs were identified based on multiple nuclei (≥ 3). Cell images were obtained using a light microscope (Leica, Germany). To measure the TRAP activity, cells were lysed with RIPA buffer following the aforementioned treatment. The TRAP activity was determined using a TRAP assay kit according to the manufacturer’s instruction. To normalize the result, the protein concentration was quantified using a BCA Protein Assay Reagent (Beyotime), and TRAP activity was expressed as U/mg protein.

### F-actin ring staining and Nrf2 immunofluorescence staining

RAW 264.7 cells were cultured in 96-well plates, followed by treatment as described in Sect. “TRAP staining and TRAP activity measurement”. After 5 d, the cells were washed with PBS, after washing cells were fixed in 4% paraformaldehyde (PFA) for 15 min, washed with PBS three times, permeabilized with 0.1% TritonX-100 for 15 min, and stained with rhodamine-phalloidin in the dark for 1 h. Nuclei were stained with DAPI for 10 min at room temperature. Finally, fluorescence images were obtained using a fluorescence microscope (Leica, Germany).

We performed Nrf2 immunofluorescence staining and nuclear translocation assays. RAW264.7 cells were seeded into 96-well plates and allowed to adhere overnight. The next day, the cells were treated with ISO (100 μM) for 2 h and then stimulated with RANKL for 30 min. After fixation with 4% PFA, the cells were blocked with 2% bovine serum albumin (diluted in PBS) for 1 h and incubated with an anti-Nrf2 antibody at 4 °C overnight. The cells were then incubated with Alexa Fluor 555-conjugated secondary antibody (Beyotime, China) for 2 h in the dark. After 3 times washing with PBS, the cells were stained with DAPI, and the final immunofluorescence images were obtained.

### Analysis using western blots

RAW264.7 cells were seeded in 6-well plates (1 × 10^5^ cells /well) and cultured overnight. After the cells adhered, to investigate early RANKL-induced signaling events, the cells were pre-treated with or without indicated concentrations ISO for 2 h, and then stimulated with 50 ng/mL RANKL for 0, 15, 30, or 60 min. To examine late stage RANLK signaling events were cultured with or without indicated concentrations of ISO and RANKL for the indicated times and extract proteins. Unstimulated cells were used as time 0 mock controls. Total protein was obtained by lysing cultured cells with RIPA lysis buffer containing PMSF and phosphatase inhibitors and cleared using centrifugation. Protein concentration was quantified using a BCA assay kit. After the sodium dodecyl sulfate–polyacrylamide gel electrophoresis (SDS-PAGE) procedure, the total protein was separated and transferred to a polyvinylidene difluoride (PVDF) membrane. After blocking the membrane with 5% skim milk for 2 h at room temperature, the membrane was probed with the corresponding primary antibody overnight at 4 °C. Next the membranes were washed thrice with Tris-buffered saline containing 0.1% Tween 20 (TBST) and then incubated with horseradish-peroxidase (HRP)-conjugated secondary antibody for 2 h. Finally, the protein was detected using an enhanced chemiluminescence reagent, and its intensity was analyzed using ImageJ software.

### Intracellular ROS assay

The level of intracellular reactive oxygen species (ROS) was detected using the ROS assay kit (Beyotime) containing the cytofluoric dye, 2’,7’‐dichlorodihydrofluorescein diacetate (DCFH-DA). RAW264.7 cells were seeded in 96-well plates, and treated with 50 ng/mL RANKL with or without different concentrations of ISO stimulation for 3 days. The cells were then incubated with the fluorescent probe containing DCFH-DA (1:1000) for 40 min in the dark. DCF fluorescence was detected using a fluorescence microscope (Leica, Germany). ROS levels were quantified using the ImageJ software.

### Quantitative real-time reverse transcription polymerase chain reaction (qRT-PCR)

RAW264.7 cells were seeded in 6-well plates, treated with different concentrations of ISO and RANKL (50 ng/mL). Five days later, total RNA was extracted from the cells using TRIzol (Thermo Fisher Scientific, USA). Complementary DNAs (cDNAs) were reverse transcribed using 1 μg of extracted total RNAs using the PrimeScript™ RT reagent Kit (TaKaRa, Japan) and used as template for subsequent qPCR reactions. The TB Green^®^ PreMix Ex Taq™ II (Takara, Japan) was used on a 7500 real-time polymerase chain reaction system (Applied Biosystems, USA). The amplification reaction conditions were: one cycle of 95 °C for 30 s, followed by 40 cycles of 95 °C for 5 s and 60 °C for 34 s. We used three different sets of internal standard primers (GAPDH, β-actin, HPRT1) to test their suitability for our study. We found that under real-time quantitative PCR conditions, the expression levels of GAPDH and β-actin were more in the range of those target genes, and the expression of GAPDH did not significantly change under any experimental conditions, making it optimal for detecting the expression of genes studied in this research. Therefore, we decided to use GAPDH as the reference gene. Primers employed for amplification are shown in Table 1. The mean cycle threshold (CT) value of the target genes was normalized to the CT value of GAPDH to obtain a ΔCT value, and analyzed using the 2^−ΔΔCt^ method. Each experiment was repeated three times.

**Table 1:** Primer sequences used for qRT-PCR analysis

Gene name	Primer sequence (5′–3′) forward	Primer sequence (5′–3′) reverse
Cathepsin K	5′- TATGACCACTGCCTTCCAATAC-3′	5′- GCCGTGGCGTTATACATACA-3′
TRAP	5′- GGAGGCTTCGATGGTGTTT- 3′	5′- CTTTCCGGTCCCAAGGATTAG-3′
MMP-9	5′- CTGGAACTCACACGACATCTT-3′	5′- TCCACCTTGTTCACCTCATTT-3′
c-Fos	5′- CGACCATGATGTTCTCGGGT -3′	5ʹ- TCGGCTGGGGAATGGTAGTA -3ʹ
NFATc1	5ʹ-AGGACCCGGAGTTCGACTT -3ʹ	5ʹ- AGGTGACACTAGGGGACACA -3ʹ
GAPDH	5′- AACAGCAACTCCCACTCTTC -3′	5′- CCTGTTGCTGTAGCCGTATT -3′
β-actin	5′- AGGCCAACCGTGAAAAGATG -3′	5′- TGGCGTGAGGGAGAGCATAG-3′
HPRT1	5′- CAAACTTTGCTTTCCCTGGTT -3′	5′- TGGCCTGTATCCAACACTTC -3′

### Rats ovariectomy experiment

All animal experimental protocols were examined and approved by the Laboratory Animal Management Committee of Xi’an Jiaotong University and were implemented following the Xi’an Jiaotong University Guide for Animal Experimentation, which conforms to the requirements of the Guide for the Care and Use of Laboratory Animals issued by the US National Institutes of Health. Thirty Sprague–Dawley (SD) female rats (8-weeks-old; 170 ± 10.3 g) were acquired from Xi’an Jiaotong University. The rats were divided into three groups: sham + Vehicle, OVX + Vehicle, and OVX + ISO (15 mg/kg) (n = 10) randomly. The three groups of rats were acclimated for one week. Under isoflurane anesthesia, bilateral ovariectomy induced OP in the OVX + Vehicle and OVX + ISO groups. In contrast, in the sham group, the rat ovaries were exposed and gently elevated without resection. After one week of recovery, OVX + ISO group rats were intraperitoneally administered 15 mg/kg/day ISO every other day for 8 weeks. Rats in the sham and OVX + Vehicle groups were intraperitoneally administered with equal volumes of the excipient (1% DMSO dissolved in physiological saline solution). ISO doses were selected based on previous research (Li et al. 2022a, b). After eight weeks of treatment, the rats were sacrificed via cervical dislocation, the femurs were removed and fixed with 4% PFA, and the left femur was subsequently collected for micro-CT scanning and histological evaluation of the right femur.

### Micro-CT analyses and histological examination

The prepared bone was transferred to a high-resolution microcomputed tomography (CT) scanner (Skyscan 1276, Belgium). The scan setting parameters were as follows: Scanning resolution 8 μm, with X-ray energy settings of 90 kV and 200 μA. After scanning, the acquired raw scan data were reconstructed in 3D using NRecon software (Bruker-microCT, Belgium) and DataViewer (Bruker-microCT, Belgium). We selected a region of interest that was 0.5 mm above the distal femoral growth plate with a height of 1 mm. Several parameters within the ROI, including bone volume/total volume (BV/TV), bone surface/bone volume (BS/BV), trabecular number (Tb.N), and trabecular separation (Tb.Sp) were measured. After micro-CT analysis, the femoral specimens were decalcified with 10% ethylenediaminetetraacetic acid (EDTA) for a sufficient period (about 1 months). Femoral specimens were dehydrated, paraffin-embedded, and sectioned using a microtome (4 μM thick). Finally, the sections were stained using the hematoxylin and eosin (H&E) and imaged under a light microscope (Leica, Germany).

### Statistical analysis

All experimental data were obtained from at least three replicate experiments, and data were expressed as mean ± SD and statistically analyzed using SPSS 24.0 software (IBM, Chicago, USA). All data were statistically analyzed using one-way ANOVA or Student’s t-test. The value of P < 0.05 was regarded as a statistically significant difference in our current study.

## Result

### Potential targets intersection of ISO with OP

**Fig. 1:**
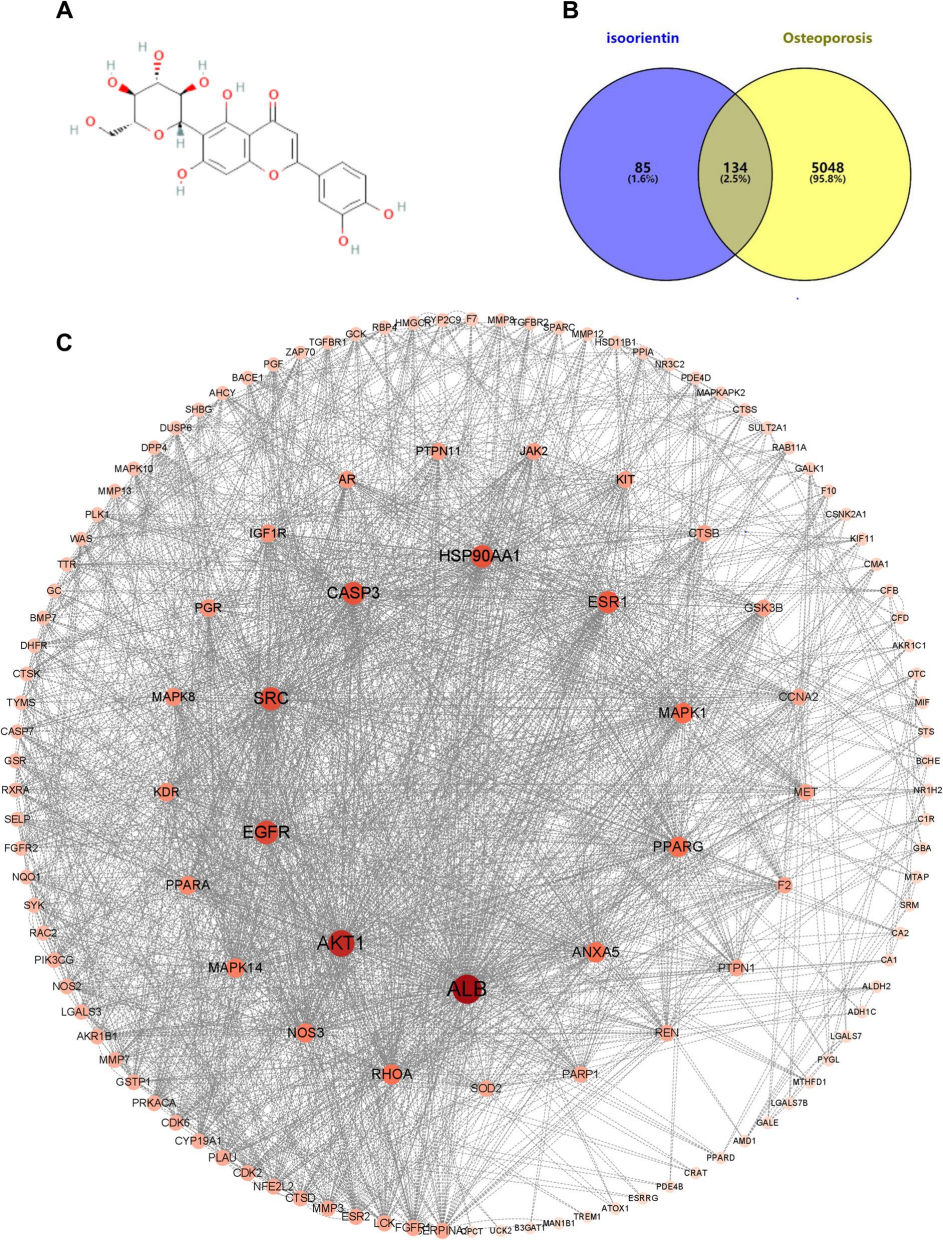
Screening of intersecting targets and construction of network diagrams of ISO and targets. **A** The chemical structure of ISO. **B** Venn diagram of cross-targeting between the ISO network and the OP gene set. **C** PPI network of potential targets. The color and size of each icon reflect the degree of the nodes of the intersection between ISO and OP

The 2D structure of ISO was downloaded from PubChem (Fig. 1A). A total of 219 potential targets of ISO were predicted using the STITCH, Swiss Target Prediction, and PharmMapper databases. After searching the OMIM, GeneCards, and TTD databases, 5182 OP-related targets were identified. By introducing all drug and disease targets into Venny 2.1.0, 134 common targets for ISO and OP were identified, and the Venn diagram was drawn (Fig. 1B).

### Protein–protein interaction network (PPI) construction

The 134 common targets were uploaded to the STRING database to obtain the network relationships of target interactions with a minimum required interaction score of > 0.4, disregarding those that did not interact with others. All data were imported into Cytoscape for the construction of a PPI network, where the color and size of the nodes represent the degree of the node. The PPI network involved 129 nodes and 1021 edges (Fig. 1C). Targets with a higher degree of relevance played an important role, with the top 10 targets being ALB, AKT1, EGFR, SRC, CASP3, HSP90AA1, ESR1, MAPK1, PPARG, and ANXA5, ranked by degree value.

### GO enrichment analysis and KEGG enrichment analysis

**Fig. 2:**
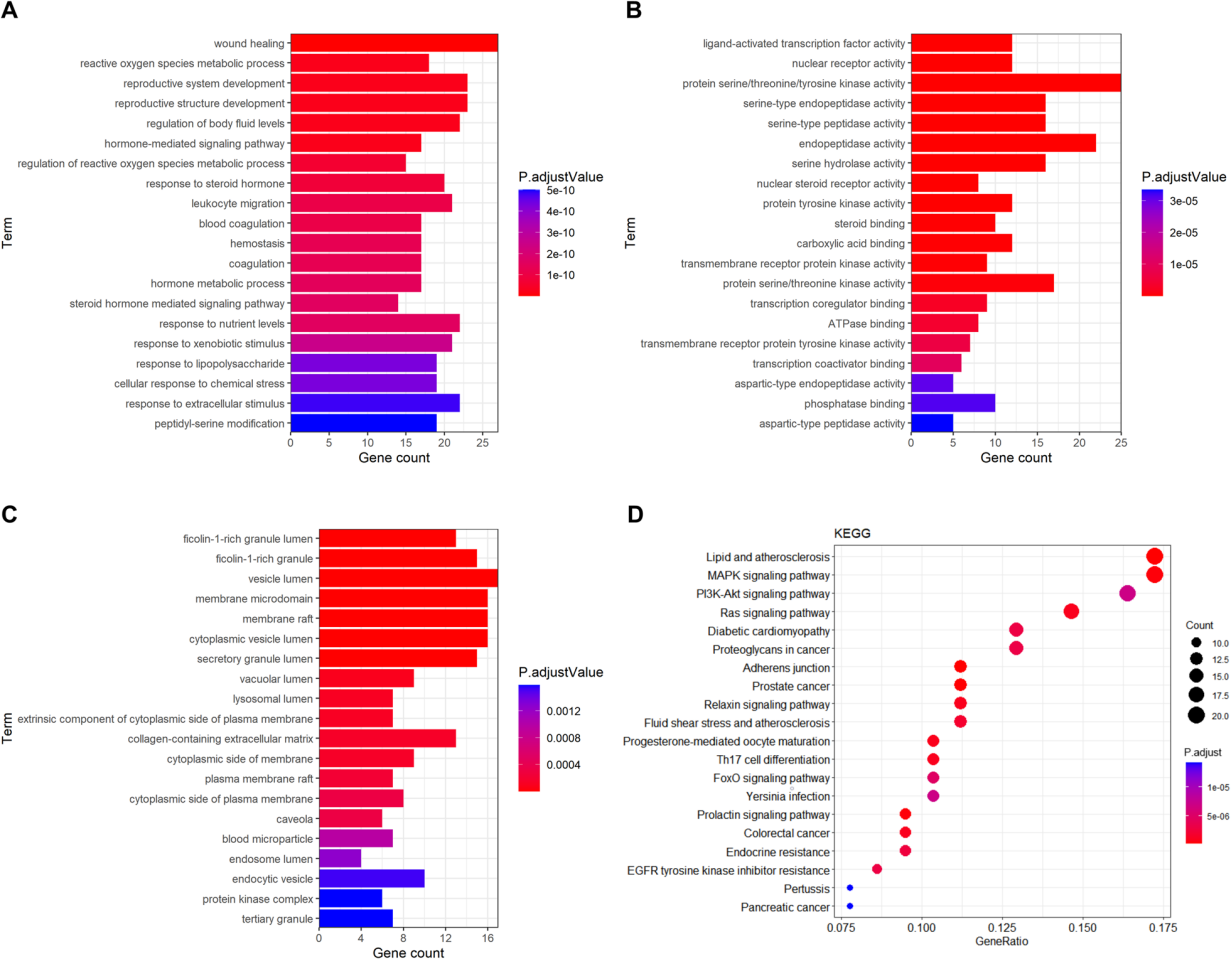
GO and KEGG pathway enrichment analysis of 134 common targets (p-value < 0.05): **A** Top 20 significantly enriched GO terms in “biological process” (BP); **B** in “molecular function” (MF) and **C** in “cellular component” (CC). **D** The bubble chart of KEGG pathway analysis (Top 20)

GO analysis was used to analyze the biological processes, cellular components, and molecular functions of the 134 common targets. The results of GO analysis showed that this set of genes was enriched in 1442 biological processes, mainly involved in wound healing, reactive oxygen metabolism processes, and the development of the reproductive system (Fig. 2A). This set of genes was enriched into 112 molecular functions, including ligand-activated transcription factor activity, nuclear receptor activity, and protein serine/threonine/tyrosine kinase activity (Fig. 2B). In addition, this set of genes was enriched into 43 cellular fractions, including the ficolin-1-rich granule lumen, ficolin-1-rich granule, and vesicle lumen (Fig. 2C).

### KEGG enrichment analysis and the enrichment analysis of target signaling pathway networks

**Fig. 3:**
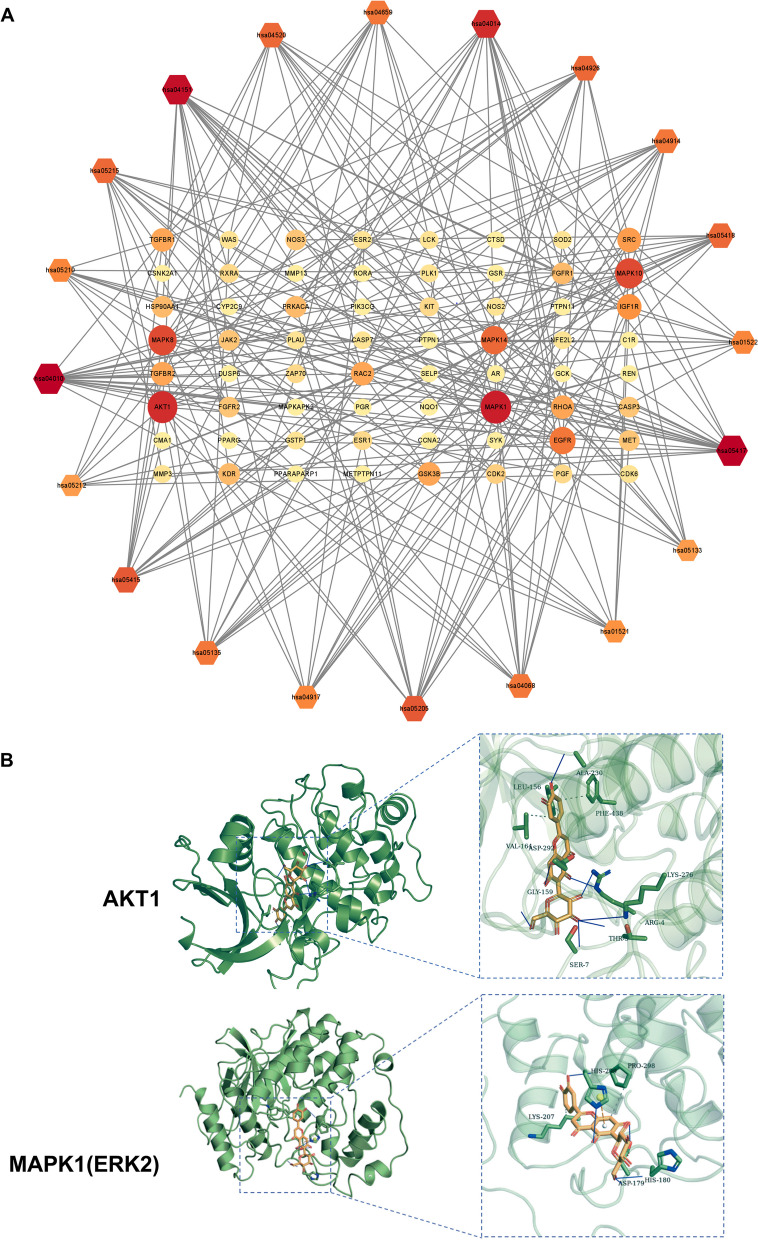
Target-pathway network of ISO against OP and simulated molecular docking of ISO on AKT1, MAPK1. **A** Target-pathway network of ISO against OP. The color and size of each icon reflect the node degree for the common targets. **B** The most optimal simulated molecular docking of ISO on AKT1, MAPK1

To further explore the potential pathways of ISO in OP treatment, KEGG analysis was performed on the 134 common targets, which enriched 131 pathways. The top 20 pathways were selected for analysis, and a bubble map was formed to enrich the KEGG pathways (Table 2, Fig. 2D). The top three KEGG pathways were the lipid and atherosclerosis, PI3K/AKT, and MAPK signaling pathways. The PI3K/AKT and MAPK pathways play critical roles in inhibiting osteoclast proliferation and differentiation, suggesting that ISO may influence the balance of bone formation by regulating the PI3K/AKT and MAPK pathways. Based on the number of targets observed in each signaling pathway, Cytoscape (3.8.2) software was used to build the target signaling pathway network (Fig. 3A). The target pathway network comprised 84 nodes and 265 edges. Combining PPI and KEGG pathways analyses and enrichment analysis of the target signaling pathway network, AKT1 and MAPK1 were identified as relatively critical targets. Among them, MAPK1 is known as ERK2 (Motta et al. 2020).

**Table 2:** KEGG pathway enrichment analysis (Top20)

Number	ID	Signaling Pathway	Enriched Gene Number	Number	ID	Signaling Pathway	Enriched Gene Number
1	hsa05417	Lipid and atherosclerosis	20	11	hsa04914	Progesterone-mediated oocyte maturation	12
2	hsa04010	MAPK signaling pathway	20	12	hsa04659	Th17 cell differentiation	12
3	hsa04151	PI3K-Akt signaling pathway	19	13	hsa04068	FoxO signaling pathway	12
4	hsa04014	Ras signaling pathway	17	14	hsa05135	Yersinia infection	12
5	hsa05415	Diabetic cardiomyopathy	15	15	hsa04917	Prolactin signaling pathway	11
6	hsa05205	Proteoglycans in cancer	15	16	hsa05210	Colorectal cancer	11
7	hsa04520	Adherens junction	13	17	hsa01522	Endocrine resistance	11
8	hsa05215	Prostate cancer	13	18	hsa01521	EGFR tyrosine kinase inhibitor resistance	10
9	hsa04926	Relaxin signaling pathway	13	19	hsa05133	Pertussis	9
10	hsa05418	Fluid shear stress and atherosclerosis	13	20	hsa05212	Pancreatic cancer	9

### Validation of compound-target interaction

AKT1 and MAPK1 were selected as key targets for molecular docking with ISO. When the binding energies of the ligand and receptor are lower, the binding conformation is more stable, and the possibility of interaction is higher. In previous studies, the binding energy ≤ − 7.0 kcal/mol indicates a strong binding between the molecule and the target (Hsin et al. 2013). The molecular docking results of ISO showed that the binding energies of ISO to the key target proteins were all less than − 7 kcal/mol, indicating that ISO has significant bindings with the receptors (Table 3). Additionally, we used PyMOL software to simulate the optimal molecular docking conformations of ISO with AKT1 and MAPK1, respectively (Fig. 3B).

**Table 3:** Analysis of simulated molecular docking of ISO with AKT1 and MAPK1

Targets	PDBID	Mode	Affinity (kcal/mol)	Targets	PDBID	Mode	Affinity (kcal/mol)
Akt1	3MVH	1	− 10.0	MAPK1	2Y9Q	1	− 8.8
		2	− 9.5			2	− 7.8
		3	− 9.3			3	− 7.7
		4	− 9.3			4	− 7.7
		5	− 9.1			5	− 7.6
		6	− 8.9			6	− 7.6
		7	− 8.9			7	− 7.5
		8	− 8.8			8	− 7.5
		9	− 8.1			9	− 7.5

### Cell viability assays

**Fig. 4:**
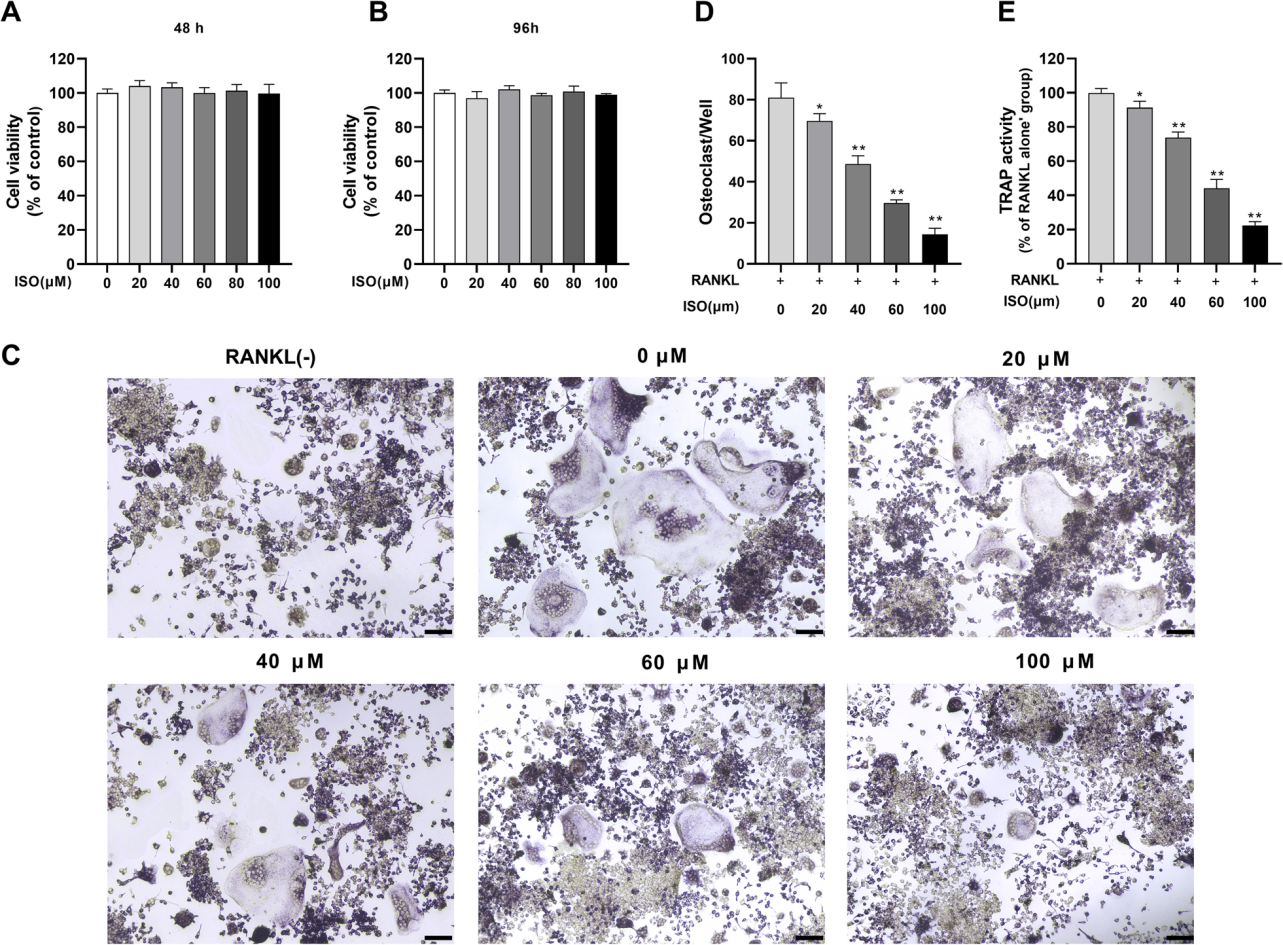
ISO dose-dependently inhibits RANKL-induced osteoclast formation and differentiation. **A**, **B** RAW264.7 cell viability as assessed by CCK-8 assay following treatment with or without indicated concentrations of ISO for 48 and 96 h. **C** RANKL-stimulated RAW264.7 cells treated with or without the indicated concentrations of ISO were stained with TRAP, and representative images were shown. **D** The number of TRAP^+^ multinucleated OCs with three or more nuclei was quantified. **E** RANKL-stimulated RAW264.7 cells treated with or without the indicated concentrations of ISO and TRAP activity was assayed. The above values were presented as mean ± standard deviation (n = 3); Scale bar = 100 μm *p < 0.05, **p < 0.01

Before assessing the impact of ISO on osteoclast formation, we determined whether ISO has a potential cytotoxic effect on RAW264.7 cells at the indicated concentrations using a CCK-8 activity assay. RAW264.7 cells were exposed to various concentrations of ISO for 48 h and 96 h. The activity of the cells did not significantly change, suggesting that ISO was not toxic to RAW264.7 cells at concentrations ≤ 100 μM (Fig. 4A, B).

### ISO inhibits RANKL-induced osteoclastogenesis and differentiation

**Fig. 5:**
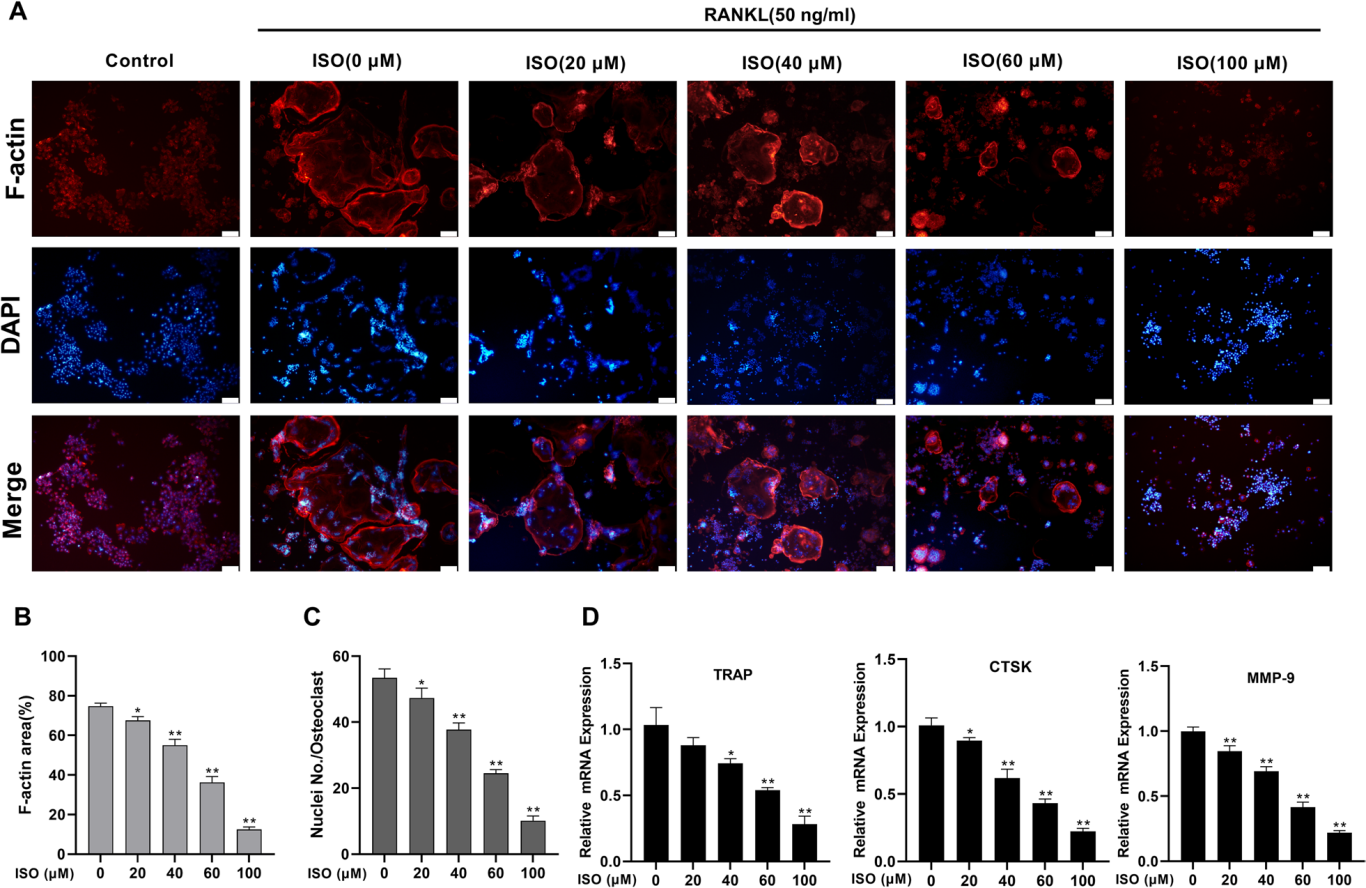
ISO attenuates RANKL-induced F-actin ring formation and inhibited the expression of osteoclast marker genes. **A** Representative fluorescence images of actin stained RAW264.7 cells stimulated with RANKL without or with different concentrations of ISO (20, 40, 60, 100 μM). The actin cytoskeleton was stained red, and the nucleus was stained blue. **B**, **C** Quantification analyses of the average cell size (based on the cell spreading area of the actin ring) and number of nuclei per osteoclast. **D** Quantitative analysis of osteoclast marker gene expression after treatment with different concentrations of ISO, was determined using qRT-PCR. Values were presented as mean ± standard deviation (n = 3); Scale bar = 100 μm; *p < 0.05, **p < 0.01

We determined that ISO did not affect the viability of RAW264.7 cells at concentrations ≤ 100 μM, and then further investigated the effect of different concentrations of ISO on RANKL-induced osteoclast formation in vitro. TRAP, a specific osteoclast marker, is important for osteoclast identification. TRAP staining and TRAP activity measurement showed that the RANKL-induced control group exhibited significant formation of TRAP-positive multinucleated OCs and a significant increase in TRAP activity. In contrast, the total number of TRAP-positive multinucleated OCs and TRAP activity were dose-dependently reduced via ISO treatment. The lowest concentration that significantly inhibited osteoclast formation was 20 μM, and the effect was most pronounced at 100 μM (Fig. 4C–E). Similar to the results of TRAP staining, treatment of OCs with ISO resulted in a dose-dependent decrease in the number and size of F-actin rings, and cells treated with 100 μM ISO were essentially monocytes compared to the multinucleated giant cells in untreated controls (Fig. 5A–C). Furthermore, to further elucidate the role of ISO in osteoclast differentiation, qRT-PCR was used to analyze the effect of ISO on the expression levels of RANKL-induced osteoclast-related genes, including TRAP, cathepsin K (CTSK), and matrix metallopeptidase‐9 (MMP-9). Activation of these genes regulates osteoclast formation and promotes osteoclast differentiation. The inhibitory effect on these genes progressively increased with the addition of ISO concentration (Fig. 5D). These results suggest that ISO had a significant inhibitory effect on osteoclast formation and differentiation.

### ISO attenuates the RANKL-activated MAPK and PI3K-AKT1 signaling pathway

**Fig. 6:**
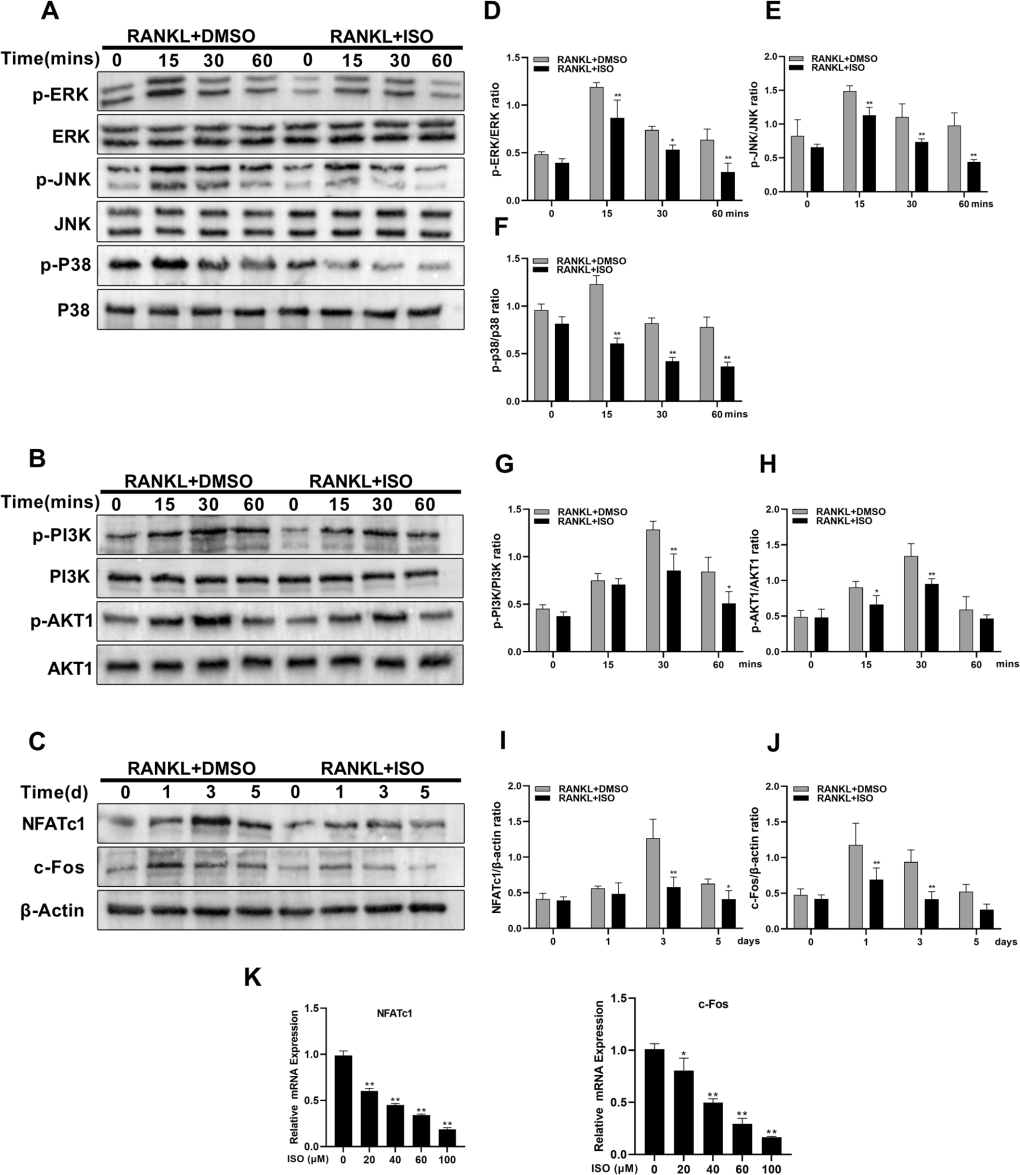
ISO inhibits RANKL-induced activation of MAPK and PI3K-AKT1 signaling cascades and down-regulated the expression of downstream c-Fos and NFATc1. **A**, **B** RAW264.7 cells were pretreated with ISO, and then total protein was extracted after stimulation with 50 ng/mL RANKL for 0, 15, 30, and 60 min. Finally, western blot analysis was performed against ERK and p-ERK, JNK and p-JNK, p38 and p-p38, p-PI3K and PI3K, p-AKT1 and AKT1. **C** RAW264.7 cells were stimulated with RANKL for the indicated times in the presence or absence of ISO at 100 μM respectively, and analyzed by western blot using specific antibodies against c-Fos, NFATc1, and β-actin. **D**–**J** Quantitative densitometric analysis of phospho-ERK relative to ERK, phospho-JNK relative to JNK, phospho-p38 relative to p38, phospho-PI3K relative to PI3K, phospho-AKT1 relative to AKT1, and NFATc1 and c-Fos relative to β-actin. **K** Quantification of relative mRNA expression of specific genes of OCs (NFATc1 and c-Fos). Values were presented as mean ± standard deviation (n = 3); *p < 0.05, **p < 0.01

Considering the signaling pathways and key targets predicted using network pharmacology, we further explored the possible role of ISO on RANKL-induced osteoclast differentiation using western blotting. We assayed for the protein levels of p-JNK, p-ERK, p-P38, p-PI3K, and p-AKT1 at 0, 15, 30, and 60 min after 50 ng/mL RANKL stimulation with or without ISO (100 μM) (Fig. 6A–B, D–H). The results showed that RANKL stimulation enhanced the phosphorylation of JNK, ERK, P38, PI3K, and AKT1 in a time-dependent manner. However, ISO significantly inhibited the phosphorylation of P38, ERK, and JNK in the MAPK signaling pathway and PI3K and AKT1.

c-Fos and NFATc1 are the main transcription factors that regulate the final differentiation of OCs, and their expression levels and corresponding protein expression levels were analyzed. RANKL gradually enhanced the expression of c-Fos and NFATc1 proteins in a time-dependent manner (Fig. 6C, I, J). However, ISO significantly inhibited the effects of RANKL on the expression of NFATc1 and c-Fos. (Fig. 6K).

### ISO reduces ROS production during RANKL-induced osteoclast formation by upregulating the expression of Nrf2 and related antioxidant enzymes

**Fig. 7:**
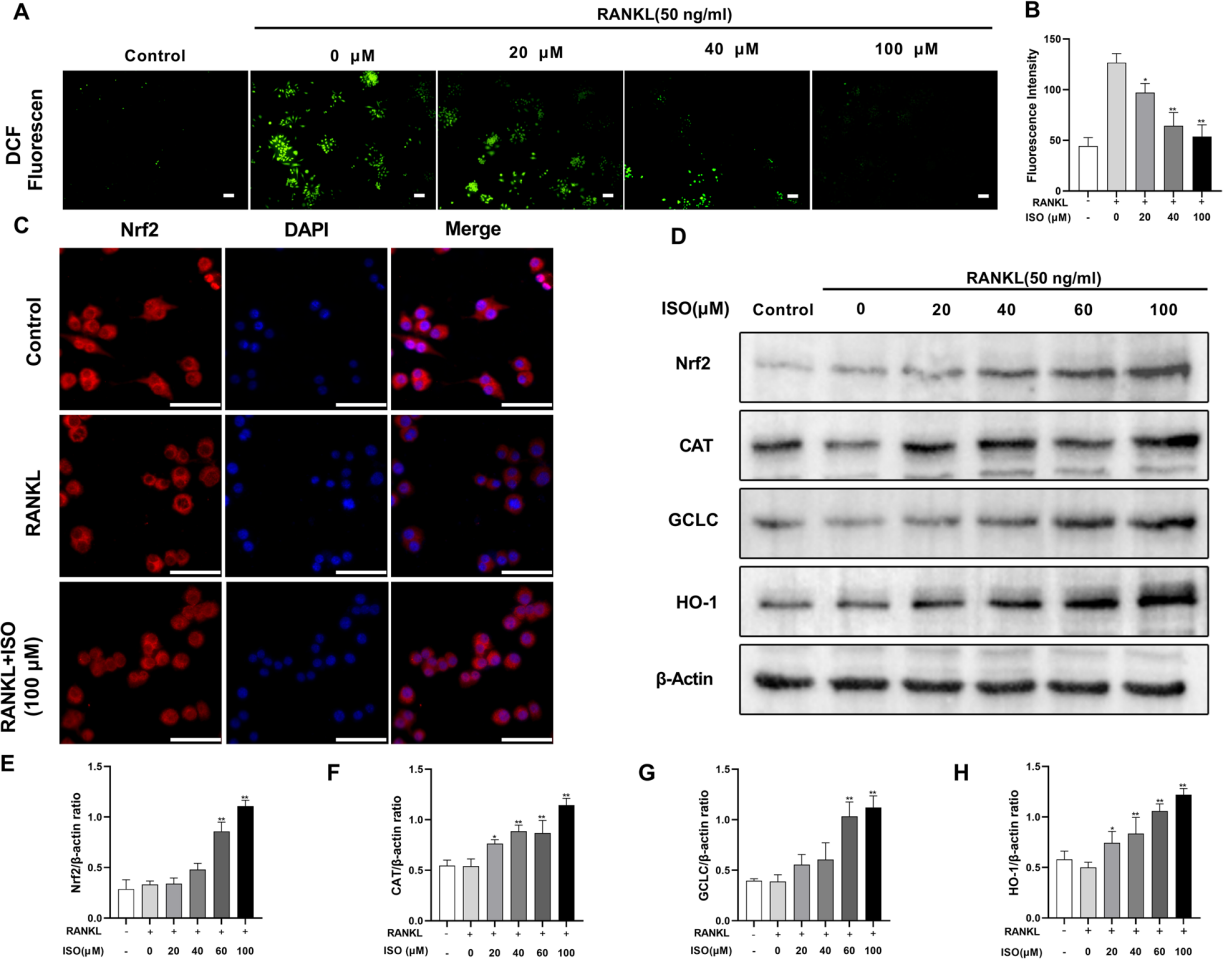
ISO decreases the level of ROS during RANKL-induced osteoclastogenesis by enhancing the expression and nuclear translocation of Nrf2 and increasing the expression of antioxidant enzymes. **A** RAW264.7 cells were stimulated with RANKL in the absence or presence of the indicated concentrations of ISO. The intracellular ROS levels were assessed by quantifying the intensity of the fluorescence of DCF, and representative confocal images were described. **B** Quantification of the average DCF fluorescence intensity of the cells in each well. **C** RAW264.7 cells were pretreated with or without 100 μM ISO and then stimulated with RANKL; Nrf2 immunofluorescence staining (red), and nuclei (blue) were labeled with DAPI. **D** Representative western blot images showing the expression of Nrf2 and several antioxidant enzymes (CAT, GCLC, and HO-1), normalized to β-actin. RAW264.7 cells, stimulated with RANKL in the absence or presence of ISO (0, 20, 40, 60, 100 μmol) for 2 d, were used for protein extraction and analysis. **E**–**H** Quantitative analysis, the band intensity of each protein was normalized to that of β-actin. Values were presented as mean ± standard deviation (n = 3); Scale bar = 100 μm; *p < 0.05, **p < 0.01

ROS are crucial regulators of OCs, affecting various osteoclast functions, including bone resorption, differentiation, proliferation, and survival (Agidigbi et al. 2019). We evaluated the changes in ROS content during osteoclast differentiation using DCFH-DA staining. The fluorescence intensity of DCF was significantly decreased by ISO treatment compared to that in the group treated with RANKL alone (Fig. 7A, B). These results suggest that ISO inhibits ROS production during RANKL-induced osteoclast formation in a dose-dependent manner. Moreover, we investigated the localization of Nrf2 in cells treated with RANKL and ISO using immunofluorescence staining (Fig. 7C). The results showed that ISO facilitated the expression and nuclear translocation of Nrf2. In addition, previous studies have shown that Nrf2 is an important factor in the regulation of osteoclast formation and activity, and its overexpression enhances the RANKL-induced elevation of antioxidant enzyme levels (Han et al. 2022). The expression levels of Nrf2-related antioxidant enzymes were analyzed using a western blot. The expression levels of Nrf2 and related antioxidant enzymes, including HO-1, CAT, and GCLC, were significantly increased after ISO treatment (within a concentration of 100 μM) in a dose-dependent manner (Fig. 7D–H).

#### ISO prevents OVX-induced bone loss in vivo

**Fig. 8:**
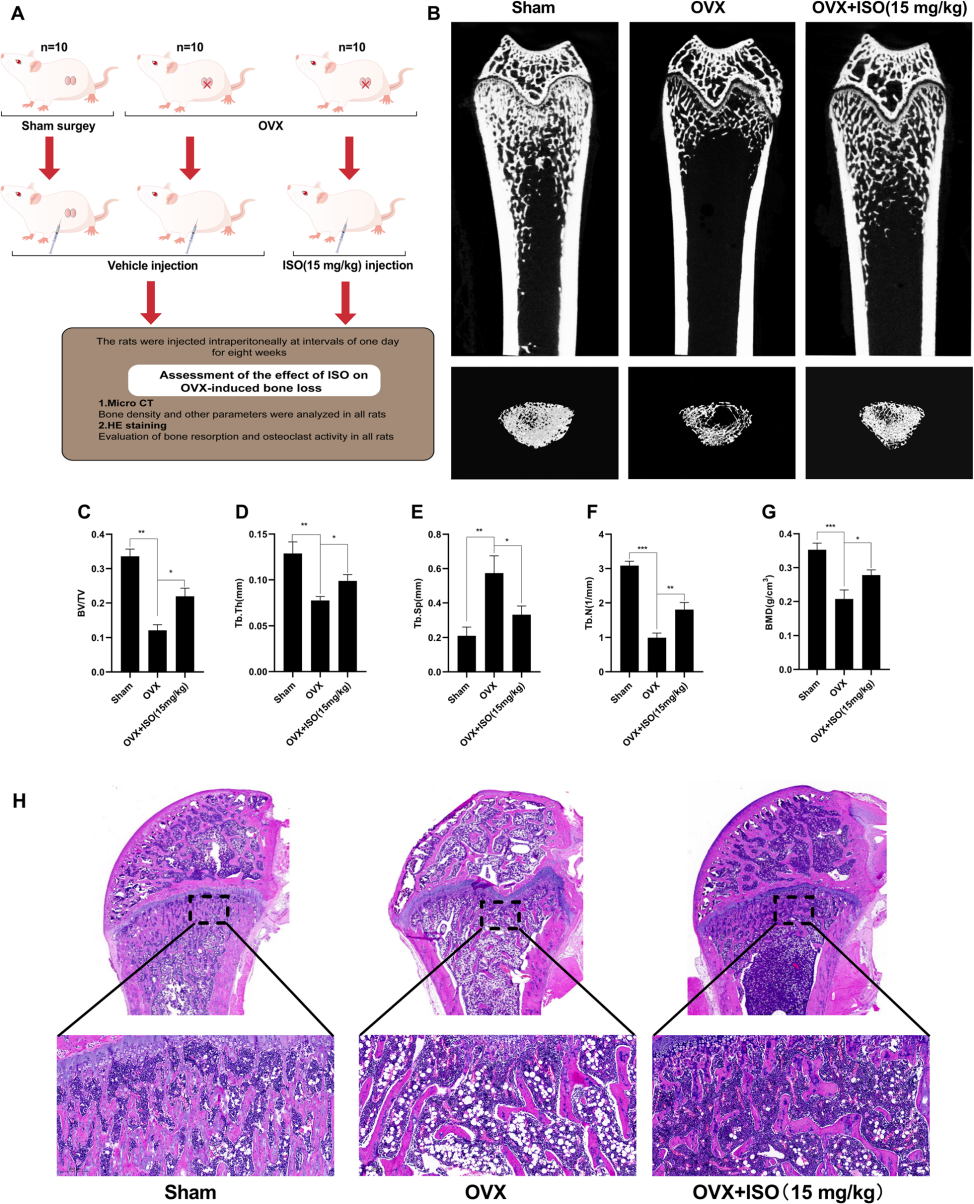
ISO prevents OVX-induced bone mass loss in vivo. **A** Flow chart representing the study design protocol to evaluate the therapeutic effect of ISO on OVX rats. **B** Representative images were acquired using micro-CT of femoral structures of different groups are shown. **C**–**G** Quantitative analyses of bone structural parameters: BV/TV, Tb.Th, Tb.Sp, Tb.N, and BMD. Values were presented as mean ± standard deviation (n = 10); **H** Representative image of H&E staining. The OVX group showed significant bone destruction, while ISO treatment significantly ameliorated OVX-induced bone loss. Scale bar = 200 μm. *p < 0.05, **p < 0.01, ***p < 0.001. BV/TV, bone volume/tissue volume; Tb.Th, trabecular thickness; Tb.Sp, trabecular separation; Tb.N, trabecular number; BMD, bone mineral density

We further investigated whether ISO could prevent bone loss in an OVX-induced rat model of OP (Fig. 8A). We analyzed the micro-CT images of the femur and evaluated the bone tissue microstructure using H&E staining. After bilateral ovariectomy, bone volume and trabeculae were significantly reduced. However, ISO administration markedly mitigated these deleterious effects, as evidenced by the increased femoral bone volume, trabeculae (Fig. 8B–H). These findings imply that ISO protects against OVX-induced bone loss primarily through osteoclast formation and activity inhibition.

## Discussion

**Fig. 9:**
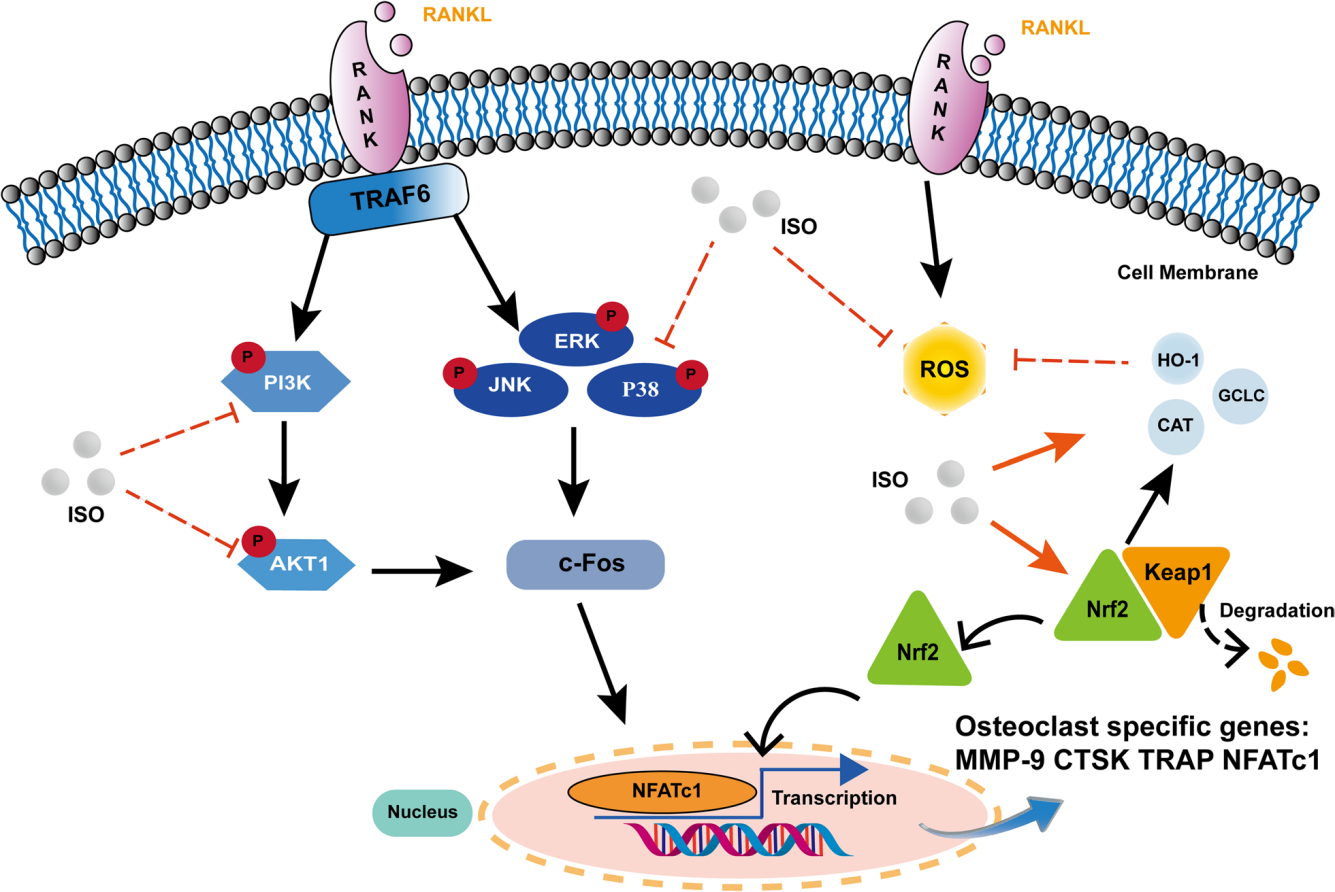
Schematic diagram of the mechanism of ISO inhibition on RANKL-induced osteoclastogenesis. Our study demonstrated that ISO could inhibit osteoclastogenesis, potentially by inhibiting the activation of the MAPK and PI3K-AKT1 signaling pathways. Additionally, ISO could reduce intracellular ROS levels by promoting the expression and nuclear translocation of Nrf2 and increasing the level of antioxidant enzymes. Eventually, the downstream activation of key signaling cascades such as c-Fos and NFATc1 transcription factors was suppressed through the above mechanism of effect. ISO, Isoorientin; RANKL, receptor activator of nuclear factor‐κB ligand; RANK, receptor activator of nuclear factor-κB; Nrf2, Nuclear factor erythroid 2‐related factor 2; Keap1, Kelch-like ECH-associated protein 1; NFATc1, nuclear factor of activated T cells 1; c‐Fos, cellular oncogene Fos; ROS, reactive oxygen species; HO‐1, haem oxygenase‐1; CAT, catalase; GCLC, γ‐glutamyl cysteine synthetase catalytic subunit; TRAP, tartrate‐resistant acid phosphatase; CTSK, cathepsin K; MMP‐9, matrix metalloproteinase‐9

Previous studies have confirmed that abnormal activation of OCs can lead to progressive bone loss, which is the pathological basis of OP, and involves the influence and regulation of multiple signaling pathways (Qu et al. 2022a, b). Representative clinical anti-OP drugs mainly include bisphosphonates, estrogens, and calcitonin. However, there are many limitations in their clinical use due to their various side effects (Li et al. 2022a, b). Consequently, searching for natural plant derivatives inhibiting OP has attracted considerable attention recently. Previous studies have shown that ISO has anti-inflammatory, and anti-tumor activities, and extremely strong antioxidant effects. In this study, network pharmacology and molecular docking analysis showed that ISO may be the active component that inhibits the formation of OCs. The main action targets were AKT1 and MAPK1, with potential signaling pathways being the PI3K-AKT, MAPK signaling pathways, and the main molecular function involved being the regulation of oxidative stress. In addition, in vitro experiments further demonstrated that ISO could effectively inhibit osteoclast formation and differentiation by regulating the PI3K-AKT1, MAPK signaling pathways, and reducing ROS production (Fig. 9). We confirmed through in vivo experiments that ISO prevented bone loss due to estrogen deficiency.

In this study, we assessed the effects of ISO on osteoclast differentiation and function. TRAP, which is highly expressed in OCs, is commonly used as a phenotypic marker. F-actin rings are the bone resorption apparatus of OCs that promote tight contact between OCs and the bone matrix and play a distinct role in osteoclast function (Lee 2018). In this study, we found through TRAP staining, TRAP activity measurement, and F-actin staining that ISO inhibits the formation, differentiation, and function of RANKL-induced OCs in a dose-dependent manner. In addition, a further noteworthy observation from our study is the significant downregulation of osteoclast-specific genes, including TRAP, CTSK, and MMP-9, in ISO-treated cells, as confirmed using qRT-PCR. Consequently, ISO may significantly inhibit osteoclast formation, differentiation, and function.

MAPK proteins are proline-directed serine/threonine kinases, including ERK, p38, and JNK (Cargnello et al. 2011; Roux et al. 2004). Two ERKs (ERK1/2) are mainly involved in the proliferation of osteoclast precursors and the formation of OCs, which are closely related to the survival of OCs (Buscà et al. 2015; Frémin et al. 2015; Miyazaki et al. 2000). In addition, phosphorylated JNK and P38 induce osteoclast differentiation and fusion, play crucial roles in osteoclastogenesis (Liu et al. 2019). PI3K is an important kinase that converts phosphatidylinositol 4,5-bisphosphate (PIP2) to phosphatidylinositol 3,4,5-trisphosphate (PIP3), which allows AKT to migrate to the membrane and become fully activated. Activated AKT is involved in cell survival, growth, and other physiological processes by activating a series of downstream intracellular proteins (Hinz et al. 2021). Chen et al. found that during osteoclast formation, both AKT1 and AKT2 genes are expressed in osteoclast precursors; however, the expression level of AKT1 is significantly higher than that of AKT2 (Chen et al. 2021). In addition, AKT1 can promote osteoblast-osteoclast coupling, and the differentiation of osteoclast precursor cells is diminished in AKT1-deficient mice (Kawamura et al. 2007; Vandoorne et al. 2010). During osteoclast formation, c-Fos, a nuclear transcription factor downstream of RANKL, promotes osteoclast formation mainly by activating the downstream factor NFATc1, which acts as the master executor of RANKL-induced osteoclast differentiation/activation and stimulates the maturation of osteoclast precursors (Takayanagi 2007; Wang et al. 2022). In this study, ISO significantly inhibited the phosphorylation of P38, ERK1/2, and JNK in the MAPK signaling pathway, as well as PI3K and AKT1. Inhibition of these signaling cascades by ISO subsequently decreased the expression of c-Fos and NFATc1, thereby inhibiting RANKL-induced osteoclast formation. This was consistent with the predicted results of the aforementioned network pharmacology analysis.

Recent studies have shown that oxidative stress is an independent risk factor that promotes osteoclast formation and differentiation, leading to skeletal diseases, especially OP (Shahriarpour et al. 2021; Zhu et al. 2022). This study indicated that ISO has an excellent antioxidant effect and could prevent redox damage to cells through an Nrf2-related mechanism, which has stronger free radical scavenging activity than antioxidants and vitamin C (Brasil et al. 2022; Yao et al. 2012). Following appropriate stimulation, a signal transmitted from the cell membrane to the cytoplasm results in the dissociation of Nrf2 from Kelch-like ECH-associated protein 1 (Keap1) and its translocated to the nucleus, where it forms a co-activation complex with small Maf proteins to activate the expression of antioxidant-related genes and products, including CAT, GCLC, and HO-1 (Qu et al. 2022a, b; Yang et al. 2022). In this study, we demonstrated that ISO dose-dependently inhibited intracellular ROS and suppressed osteoclast differentiation by upregulating the expression of Nrf2, promoting its nuclear translocation, and increasing the expression of related antioxidant enzymes.

Considering the in vitro inhibitory effects of ISO on RANKL-mediated osteoclast differentiation and function, we expanded our investigation to an in vivo context using an OVX rat model. Evaluation using micro-CT scanning along with hematoxylin and eosin (H&E) staining of femoral specimens showed a reduction in bone loss and an increase in trabecular bone after ISO treatment. These observations demonstrate the protective role of ISO against osteoclastogenesis and bone loss in OVX rats, highlighting its potential therapeutic applications in osteoclast-related diseases.

In summary, we demonstrated that ISO could inhibit RANKL-induced osteoclast formation and activity by suppressing the activation of the MAPK and PI3K/AKT1 signaling pathways in vitro and protecting against OVX-induced bone loss in vivo. Furthermore, ISO suppressed RANKL-induced ROS production by upregulating Nrf2 expression and activating intracellular antioxidant enzymes, inhibiting the downstream activation of key signals including c-Fos and NFATc1 transcription factors. However, our experiment also has certain limitations. Firstly, we need to further investigate and expand on the relevant mechanisms. Second, previous studies have shown that the regulation of AKT1 also affects osteoblastogenesis (Mukherjee et al. 2012; Tang et al. 2021). Therefore, to determine whether ISO impacts osteoblast proliferation and differentiation, more experiments are needed to prove this hypothesis. Third, more in-depth research into the in vivo safety of ISO is required. Altogether, the results of this study suggest that ISO is a potential drug for the treatment of osteoclast-related diseases and provide relevant evidence for this.

## Data Availability

The datasets used to support the findings of this study are available from the corresponding author upon request.

## Abbreviations

ISO: Isoorientin
RANK: Receptor activator of nuclear factor-κB
RANKL: Receptor activator of nuclear factor-κB ligand
ROS: Reactive oxygen species
OVX: Ovariectomy
Nrf2: Nuclear factor erythroid 2‐related factor 2
TRAP: Tartrate‐resistant acid phosphatase
TRAF6: Tumor necrosis factor receptor-associated factor 6
MAPK: Mitogen‐activated protein kinase
PI3K: Phosphatidylinositol 3 kinases
c-Fos: Cellular oncogene Fos
NFATc1: Nuclear factor of activated T cells 1
PPI: Protein–protein interaction network
GO: Gene ontology
KEGG: Kyoto Encyclopedia of Genes and Genomes
CTSK: Cathepsin K
MMP-9: Matrix metallopeptidase‐9
HO-1: Haem oxygenase‐1
CAT: Catalase
GCLC: γ‐Glutamyl cysteine synthetase catalytic subunit
Keap1: Kelch-like ECH-associated protein 1

## References

[CR1] Agidigbi TS, Kim C (2019). Reactive oxygen species in osteoclast differentiation and possible pharmaceutical targets of ROS-mediated osteoclast diseases. Int J Mol Sci.

[CR2] Anilkumar K, Reddy GV, Azad R, Yarla NS, Dharmapuri G, Srivastava A (2017). Evaluation of anti-inflammatory properties of isoorientin isolated from tubers of. Oxid Med Cell Longev.

[CR3] Brasil FB, de Almeida FJS, Luckachaki MD, Dall'Oglio EL, de Oliveira MR (2022). A pretreatment with isoorientin attenuates redox disruption, mitochondrial impairment, and inflammation caused by chlorpyrifos in a dopaminergic cell line: involvement of the Nrf2/HO-1 axis. Neurotox Res.

[CR4] Buscà R, Christen R, Lovern M, Clifford AM, Yue J-X, Goss GG (2015). ERK1 and ERK2 present functional redundancy in tetrapods despite higher evolution rate of ERK1. BMC Evol Biol.

[CR5] Cargnello M, Roux PP (2011). Activation and function of the MAPKs and their substrates, the MAPK-activated protein kinases. Microbiol Mol Biol Rev.

[CR6] Chen X, Chen W, Aung ZM, Han W, Zhang Y, Chai G (2021). LY3023414 inhibits both osteogenesis and osteoclastogenesis through the PI3K/Akt/GSK3 signalling pathway. Bone Joint Res.

[CR7] Elson A, Anuj A, Barnea-Zohar M, Reuven N (2022). The origins and formation of bone-resorbing osteoclasts. Bone.

[CR8] Frémin C, Saba-El-Leil MK, Lévesque K, Ang SL, Meloche S (2015). Functional redundancy of ERK1 and ERK2 MAP kinases during development. Cell Rep.

[CR9] Gao L, Zhang SQ (2022). Antiosteoporosis effects, pharmacokinetics, and drug delivery systems of icaritin: advances and prospects. Pharmaceuticals (basel).

[CR10] Gong W, Chen X, Shi T, Shao X, An X, Qin J (2021). Network pharmacology-based strategy for the investigation of the anti-osteoporosis effects and underlying mechanism of zhuangguguanjie formulation. Front Pharmacol.

[CR11] Han J, Yang K, An J, Jiang N, Fu S, Tang X (2022). The role of NRF2 in bone metabolism—friend or foe?. Front Endocrinol (lausanne).

[CR12] Hinz N, Jücker M (2021). AKT in bone metastasis of solid tumors: a comprehensive review. Cancers.

[CR13] Hopkins AL (2008). Network pharmacology: the next paradigm in drug discovery. Nat Chem Biol.

[CR14] Hsin K-Y, Ghosh S, Kitano H (2013). Combining machine learning systems and multiple docking simulation packages to improve docking prediction reliability for network pharmacology. PLoS ONE.

[CR15] Huang C, Zheng C, Li Y, Wang Y, Lu A, Yang L (2014). Systems pharmacology in drug discovery and therapeutic insight for herbal medicines. Brief Bioinform.

[CR16] Kawamura N, Kugimiya F, Oshima Y, Ohba S, Ikeda T, Saito T (2007). Akt1 in osteoblasts and osteoclasts controls bone remodeling. PLoS ONE.

[CR17] Kitaura H, Marahleh A, Ohori F, Noguchi T, Shen WR, Qi J (2020). Osteocyte-related cytokines regulate osteoclast formation and bone resorption. Int J Mol Sci.

[CR18] Lee BS (2018). Myosins in osteoclast formation and function. Biomolecules.

[CR19] Li S, Liu H, Lin Z, Li Z, Chen Y, Chen B (2022). Isoorientin attenuates doxorubicin-induced cardiac injury via the activation of MAPK, Akt, and Caspase-dependent signaling pathways. Phytomedicine.

[CR20] Li Y, Li L, Li X, Luo B, Ye Q, Wang H (2022). A mechanistic review of Chinese medicine polyphenols on bone formation and resorption. Front Pharmacol.

[CR21] Liu Y, Wang C, Wang G, Sun Y, Deng Z, Chen L (2019). Loureirin B suppresses RANKL-induced osteoclastogenesis and ovariectomized osteoporosis via attenuating NFATc1 and ROS activities. Theranostics.

[CR22] Lorenzo J (2017). The many ways of osteoclast activation. J Clin Invest.

[CR23] Miyazaki T, Katagiri H, Kanegae Y, Takayanagi H, Sawada Y, Yamamoto A (2000). Reciprocal role of ERK and NF-kappaB pathways in survival and activation of osteoclasts. J Cell Biol.

[CR24] Motta M, Pannone L, Pantaleoni F, Bocchinfuso G, Radio FC, Cecchetti S (2020). Enhanced MAPK1 function causes a neurodevelopmental disorder within the RASopathy clinical spectrum. Am J Hum Genet.

[CR25] Mukherjee A, Rotwein P (2012). Selective signaling by Akt1 controls osteoblast differentiation and osteoblast-mediated osteoclast development. Mol Cell Biol.

[CR26] Nannan X, Liyang J, Qiyan LI (2023). Potential of natural medicines for treatment of osteoporosis: a narrative review. J Tradit Chin Med.

[CR27] Nor Muhamad ML, Ekeuku SO, Wong S-K, Chin K-Y (2022). A scoping review of the skeletal effects of naringenin. Nutrients.

[CR28] Park-Min K-H, Lorenzo J (2022). Osteoclasts: other functions. Bone.

[CR29] Qu Z, An H, Feng M, Huang W, Wang D, Zhang Z (2022). Urolithin B suppresses osteoclastogenesis via inhibiting RANKL-induced signalling pathways and attenuating ROS activities. J Cell Mol Med.

[CR30] Qu Z, Zhang B, Kong L, Gong Y, Feng M, Gao X (2022). Receptor activator of nuclear factor-κB ligand-mediated osteoclastogenesis signaling pathway and related therapeutic natural compounds. Front Pharmacol.

[CR31] Roux PP, Blenis J (2004). ERK and p38 MAPK-activated protein kinases: a family of protein kinases with diverse biological functions. Microbiol Mol Biol Rev.

[CR32] Shahriarpour Z, Nasrabadi B, Hejri-Zarifi S, Shariati-Bafghi SE, Yousefian-Sanny M, Karamati M (2021). Oxidative balance score and risk of osteoporosis among postmenopausal Iranian women. Arch Osteoporos.

[CR33] Takayanagi H (2007). Osteoimmunology: shared mechanisms and crosstalk between the immune and bone systems. Nat Rev Immunol.

[CR34] Tang Y, Mo Y, Xin D, Xiong Z, Zeng L, Luo G (2021). Regulation of osteoblast autophagy based on PI3K/AKT/mTOR signaling pathway study on the effect of β-ecdysterone on fracture healing. J Orthop Surg Res.

[CR35] Vandoorne K, Magland J, Plaks V, Sharir A, Zelzer E, Wehrli F (2010). Bone vascularization and trabecular bone formation are mediated by PKB alpha/Akt1 in a gene-dosage-dependent manner: in vivo and ex vivo MRI. Magn Reson Med.

[CR36] Wang X, Wang Z, Zheng J, Li S (2021). TCM network pharmacology: a new trend towards combining computational, experimental and clinical approaches. Chin J Nat Med.

[CR37] Wang MY, An MF, Fan MS, Zhang SS, Sun ZR, Zhao YL (2022). FAEE exerts a protective effect against osteoporosis by regulating the MAPK signalling pathway. Pharm Biol.

[CR38] Xu WT, Shen GN, Li TZ, Zhang Y, Zhang T, Xue H (2020). Isoorientin induces the apoptosis and cell cycle arrest of A549 human lung cancer cells via the ROS-regulated MAPK, STAT3 and NF-κB signaling pathways. Int J Oncol.

[CR39] Yang K, Cao F, Xue Y, Tao L, Zhu Y (2022). Three classes of antioxidant defense systems and the development of postmenopausal osteoporosis. Front Physiol.

[CR40] Yao H, Chen Y, Shi P, Hu J, Li S, Huang L (2012). Screening and quantitative analysis of antioxidants in the fruits of Livistona chinensis R. Br using HPLC-DAD-ESI/MS coupled with pre-column DPPH assay. Food Chem.

[CR41] Yuan L, Wu Y, Ren X, Liu Q, Wang J, Liu X (2014). Isoorientin attenuates lipopolysaccharide-induced pro-inflammatory responses through down-regulation of ROS-related MAPK/NF-κB signaling pathway in BV-2 microglia. Mol Cell Biochem.

[CR42] Zhu C, Shen S, Zhang S, Huang M, Zhang L, Chen X (2022). Autophagy in bone remodeling: a regulator of oxidative stress. Front Endocrinol (lausanne).

[CR43] Ziqubu K, Dludla PV, Joubert E, Muller CJF, Louw J, Tiano L (2020). Isoorientin: a dietary flavone with the potential to ameliorate diverse metabolic complications. Pharmacol Res.

